# The Contribution of Neutrophils and NETs to the Development of Type 1 Diabetes

**DOI:** 10.3389/fimmu.2022.930553

**Published:** 2022-07-06

**Authors:** Alessandra Petrelli, Sarah K. Popp, Riho Fukuda, Christopher R. Parish, Emanuele Bosi, Charmaine J. Simeonovic

**Affiliations:** ^1^ San Raffaele Diabetes Research Institute, Istituto di Ricovero e Cura a Carattere Scientifico (IRCCS) Ospedale San Raffaele, Milan, Italy; ^2^ Immunology and Infectious Disease Division, The John Curtin School of Medical Research, The Australian National University, Canberra, ACT, Australia; ^3^ Department of Medicine, Tokyo Medical and Dental University, Bunkyo City, Tokyo, Japan; ^4^ Genome Sciences and Cancer Division, The John Curtin School of Medical Research, The Australian National University, Canberra, ACT, Australia; ^5^ Department of Medicine, San Raffaele Vita Salute University, Milan, Italy

**Keywords:** neutrophils, NETs, platelet-neutrophil aggregates, Type 1 diabetes, islets, exocrine dysfunction

## Abstract

Type 1 diabetes (T1D) is an autoimmune disease resulting from the destruction of insulin-producing beta cells in pancreatic islets. T lymphocytes are the claimed pathogenic effectors but abnormalities of other immune cell types, including neutrophils, also characterize T1D development. During human T1D natural history, neutrophils are reduced in the circulation, while accumulate in the pancreas where release of neutrophil extracellular traps (NETs), or NETosis, is manifest. Recent-onset T1D patients also demonstrate activated circulating neutrophils, associated with a unique neutrophil gene signature. Neutrophils can bind to platelets, leading to the formation of platelet-neutrophil aggregates (PNAs). PNAs increase in the circulation during the development of human T1D and provide a mechanism for neutrophil activation and mobilization/recruitment to the pancreas. In non-obese diabetic or NOD mice, T1D autoimmunity is accompanied by dynamic changes in neutrophil numbers, activation state, PNAs and/or NETosis/NET proteins in the circulation, pancreas and/or islets. Such properties differ between stages of T1D disease and underpin potentially indirect and direct impacts of the innate immune system in T1D pathogenesis. Supporting the potential for a pathogenic role in T1D, NETs and extracellular histones can directly damage isolated islets *in vitro*, a toxicity that can be prevented by small polyanions. In human T1D, NET-related damage can target the whole pancreas, including both the endocrine and exocrine components, and contribute to beta cell destruction, providing evidence for a neutrophil-associated T1D endotype. Future intervention in T1D could therefore benefit from combined strategies targeting T cells and accessory destructive elements of activated neutrophils.

## Introduction to NETs in T1D

Type 1 diabetes is an autoimmune disease in which insulin-producing beta cells in pancreatic islets undergo immune destruction, ultimately resulting in hyperglycemia. The detection of beta cell autoantigen-reactive T cells in peripheral blood (1, 2) and islet inflammation (insulitis) (3–5) together with the absence of autoantibody-mediated beta cell damage (6, 7), have supported the concept that T1D in humans is T cell-mediated. However, recent evidence has suggested that cells of the innate immune system, particularly neutrophils, also participate in T1D development in at least a subset of individuals. Similarly, differences in islet- infiltrating immune cell populations have been identified in children who develop clinical T1D at different ages (8). These and other studies support the concept that T1D disease is heterogeneous and that disparities between the disease process in different individuals relate to distinct endotypes (9–12).

Studies including the NOD mouse model of T1D, have confirmed multiple roles for activated/NETosing neutrophils in T1D pathogenesis, with the mechanism of neutrophil activation appearing to differ between disease initiation and progression. Although circulating NETs are elevated in recent-onset human T1D (13), the detection of NETs in the pancreas is infrequent, possibly due to their rapid degradation by locally produced exocrine DNAse (14). As a consequence, NET products, e.g., myeloperoxidase or MPO, neutrophil elastase (NE) and citrullinated histones (CitH3), are commonly used as surrogate markers of NETosis and provide a focus for elucidating the function(s) of neutrophils in T1D. Strongly supporting a pathogenic role for neutrophils/NETs in T1D, circulating levels of indirect markers of neutrophil activation (e.g., PNAs) (15) and NET proteins (e.g., NE) (13) follow a dynamic pattern throughout T1D development, largely mirroring levels in islets in NOD mice at different stages of disease progression (15) or negatively correlating with glycemic control/beta cell function in humans (13). Surprisingly, studies of T1D human pancreas samples have also revealed neutrophil infiltration and NETosis in exocrine pancreatic tissue (16, 17), exposing T1D as a complex immune disease of the pancreas which extends beyond autoimmune damage of islet beta cells (18).

## Role for Neutrophils, NETs and PNAs in the Development of T1D

### Human T1D

Both innate and adaptive immunity participate in T1D pathogenesis in humans. Neutrophils, the most abundant phagocytic cell in human blood (19), display heterogeneous functions and flexibility, developing different profiles in response to different disorders, including autoimmune diseases (20). A role for neutrophils has been consistently shown in human T1D (15–17, 21). Circulating neutrophil counts are reduced to the lower limits of the normal range in patients with recent-onset T1D; first‐degree relatives of T1D patients with 2 or more autoantibodies (i.e., pre-symptomatic or pre-T1D) also exhibit a lower circulating neutrophil count which parallels the development of beta cell dysfunction (16). This neutrophil abnormality is not attributed to neutrophil cell death, impaired differentiation or targeting by anti-neutrophil antibodies (16), suggesting that the fate of neutrophils in pre-T1D may be to exit the circulation and infiltrate the pancreas. The decrease in circulating neutrophil counts is stable for up to 1 year after the onset of T1D (22), with normal levels returning thereafter (13).

Neutrophils from T1D and pre‐symptomatic subjects display an interferon (IFN) pro‐inflammatory signature (17), resembling other autoimmune diseases such as rheumatoid arthritis (23) and systemic lupus erythematosus (SLE) (24). Neutrophils from T1D patients are altered in their phenotype and function. Post-T1D onset, they display reduced migration and chemotaxis (25), paralleling the sharp decline in circulating PNA levels (15). They also exhibit impaired phagocytic capacity, leading to reduced clearance of bacteria (26) and to hyperglycemia-dependent increased susceptibility to infections (27).

Activated neutrophils release a variety of serine proteases from intracellular granules, including NE and proteinase 3 (PR‐3), which help to eliminate microorganisms and regulate immune responses during inflammation (28). Circulating levels of NE and PR3 are increased in patients with T1D and correlate with numbers and titres of autoantibodies (13). Furthermore, MPO, an enzyme involved in microbial killing (29), is similarly increased, regardless of T1D duration (30). Despite the uncertain mechanism underlying neutrophil-mediated development of islet autoimmunity in humans, NE, PR‐3 and MPO may each contribute to T1D pathogenesis, possibly targeting beta cells, resident pancreatic cells, or have other roles, as revealed in NOD mice.

Besides phagocytosis and degranulation, neutrophils can damage host tissues by the release of NETs. NETs are extracellular web-like structures composed of cytosolic and granule proteins assembled on decondensed chromatin (31). Normally the function of NETs is to neutralize and kill microorganisms. If dysregulated, NETs can contribute to the pathogenesis of immune-mediated diseases (32). Enhanced NETs have been described in the circulation of T1D patients (13), where they display an altered composition compared to healthy individuals and induce Th1 polarization (33). Notably, peptidyl arginine deiminase‐4 (PAD4), the enzyme responsible for histone citrullination and NET formation (34), is increased in neutrophils of patients with T1D and T2D, leading to increased NETosis upon stimulation (35). While increased NET formation is triggered by hyperglycemia after T1D-onset, NETosis also occurs in pre-T1D individuals, as suggested by deposition of NET products (CitH3, MPO) in the pancreas (17).

Altered numbers and function of neutrophils have been described not only in the circulation, but also in the pancreas of patients with T1D. Indeed we have identified neutrophils and decondensed DNA decorated with MPO and citrullinated histones, indicating the presence of pancreas-residing neutrophils releasing NETs, in both pre-T1D and T1D individuals (16, 17). Furthermore, we have recently demonstrated that extracellular histones damage human islets *in vitro* (15), providing a putative mechanism for neutrophil-mediated islet cell damage. Of note, methyl cellobiose sulfate (mCBS), a small polyanionic drug that neutralizes the high positive charge of histones, prevented islet cell death (15, 36). Thus, mCBS could offer potential, in combination with other agents, for delaying T1D progression. These findings corroborate the hypothesis that neutrophils play an ancillary role in the development of islet autoimmunity and beta cell damage during the pathogenesis of T1D.

How neutrophils are activated to release NETs in T1D is not completely understood. Activated platelets can bind neutrophils through an interaction between platelet cell surface CD62P (P-selectin) and neutrophil P-selectin binding glycoprotein ligand 1 (PSGL1) (32, 37), which triggers the release of NETs (32, 38). In this setting, initial platelet activation could occur *via* the binding of citrullinated histones to platelet TLR2/TLR4 (39), supporting a vicious cycle of platelet and neutrophil activation, or potentially by autoantigen-IgG immune complexes binding to platelet FcγRIIA (40). Platelets have been shown to direct neutrophil trafficking (41) and P-selectin interactions with PSGL-1 at the vessel wall guide neutrophil extravasation (42). We have recently shown that PNAs are elevated in some pre-T1D individuals and new-onset T1D patients, and that a lower circulating neutrophil count correlates with a higher proportion of PNAs (15). Furthermore, we revealed platelet hyperreactivity in PNAs preceding T1D onset (15). These data support the hypothesis that neutrophils are reduced in the circulation prior to the development of T1D because they are bound to activated platelets, with this interaction promoting neutrophil activation, trafficking to the pancreas, subsequent NET release and islet damage/beta cell impairment. Altogether, these data provide evidence for the potential contribution of innate immune responses to T1D disease progression in humans.

### NOD Mouse Model of T1D

Studies in the NOD mouse model of autoimmune T1D have revealed possible ancillary and effector roles for neutrophils in the initiation and progression of T1D disease. During the initiation of T1D autoimmunity, a transient elevation in neutrophil numbers in the islets of 3-4 week old NOD mice (43, 44) was accompanied by the activation of neutrophils by antibodies against beta cell DNA (anti-DNA Ig), CRAMP (anti-microbial peptide) release and occasional peri-islet NETosis (43). The *in vivo* depletion of neutrophils and other leukocyte populations in young NOD mice prevented later T1D-onset and were considered to define a *local accessory role* for neutrophils in activating plasmacytoid dendritic cells (DCs) and the local production of IFNα. Such neutrophil interactions may indirectly promote autoantigen presentation by conventional DCs in pancreatic lymph nodes, activating autoreactive T cells and initiating early autoimmune damage of beta cells (43).

In contrast, evidence for prolonged *systemic* neutrophil activation was found in longitudinal studies of NOD mice from 2-30 weeks of age. NE and PR3 activities were elevated in the circulation for > 10 weeks in only NOD mice which progressed to T1D (13). These findings suggested that circulating neutrophils may contribute to both the initiation and progression of T1D disease, potentially either by degranulation in the bloodstream and release of granule-derived enzymes or from the expulsion of such enzymes during NETosis. By treating young NOD mice with pharmacological or genetically engineered inhibitors of NE, Shu et al. reported NE-induced islet inflammation throughout T1D development, inferring an *indirect role* for activated neutrophils in autoimmune damage of beta cells (45). NOD mouse models have further identified an inter-relationship between certain gut microbiota, increased gut permeability, neutrophil numbers/NETosis and T1D (46, 47). In particular, gut leakage of abnormal microbiota/lipopolysaccharide (LPS) has been reported to stimulate local NETosis in NOD mice, resulting in NET-assisted activation of enteric CD4 T cells which, upon migration, can enhance T1D autoimmunity (47). Such studies provide a possible *indirect* link between neutrophils/NETs and adaptive immunity in T1D.

**Figure 1:**
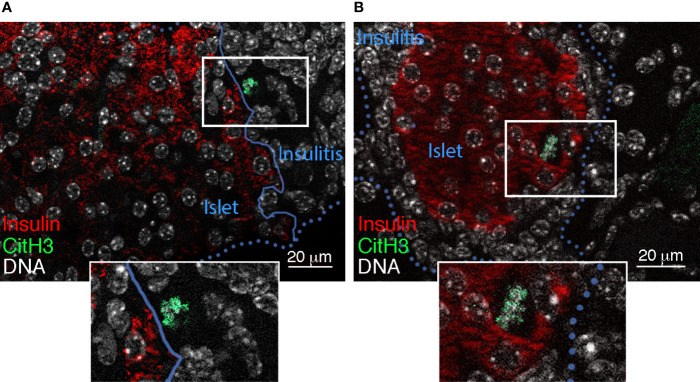
Islet-associated CitH3 in NOD pancreas. Immunofluorescence detection of insulin (red) (48) and CitH3 (green) (15) (using AF488 donkey anti-rabbit Ig) in pancreas sections from **(A)** 4 week-old and **(B)** 10-12 week-old female NOD mice. Blue unbroken and dotted lines define the islet border in **(A)** and insulitis, respectively. DNA is labelled using Hoechst 33342 (white).

Similar to studies of human pancreata (16, 17) and young NOD mice (44), we recently reported a significant increase in the immunohistochemical localization of *islet-associated* NET products (MPO and CitH3) in the pancreas of adult pre-T1D (10-12 weeks of age) and T1D-onset NOD mice (15) (**Figure 1**). In parallel, calf thymus histones and NETs were found to be toxic for isolated mouse islets, with NET- as well as histone-induced islet damage being prevented by short-term culture with mCBS (15, 36). Collectively, these findings suggested that citrullinated histones released from NETosing neutrophils could *directly* contribute to islet/beta cell damage during T1D development, most likely by inducing pores in the plasma membrane of islet/beta cells (15, 36, 49, 50).

The reason underlying the recruitment of neutrophils to the pancreas during the *initiation* of T1D autoimmunity in very young NOD mice has been attributed to a physiological wave of beta cell apoptosis that occurs during pancreas differentiation (51). This process is envisaged to prompt an acute inflammatory response to islets, triggered especially by chemokines (CXCL1, CXCL2) released from damaged beta cells and/or intra-islet macrophages (43, 52). Endothelial cells inadvertently injured during this early inflammatory response and neutrophil (PSGL1)-endothelial (P-Selectin or CD62P) interactions could facilitate neutrophil influx into the pancreas (44) and the appearance of activated neutrophils in the islet microenvironment (43).

Levels of PNAs are elevated in the circulation of young NOD mice at 4 weeks as well as adult NOD mice at 10-12 weeks (pre-T1D) and at T1D-onset, largely mimicking increased circulating PNAs in at-risk Aab-positive and T1D-onset children (15). PNA levels in NOD islets were similarly increased at 10-12 weeks and T1D-onset, correlating with a significant rise in the proportion of islets that were MPO- or CitH3-positive. High expression of CD62P on platelets within NOD mouse PNAs suggested that CD62P (platelet)-PSGL1 (neutrophil) interactions are key to PNA formation and neutrophil activation, similar to human PNAs. Subsequently, neutrophil mobilization to pancreatic islets, most likely occurs *via* platelet-derived proplatelet binding protein (PPBP) which undergoes cleavage to form CXCL7/NAP2 (a neutrophil -activating and - recruiting chemokine) (53–55) and has been found elevated in T1D human blood by serum proteome analysis (56). Ultimately this pathway results in islet-associated NETosis (15). Thus, stimulation of neutrophils by activated platelets could provide a supplementary mechanism driving the contribution of neutrophils to the *initiation* of T1D disease. Thereafter, a pivotal role has been proposed for platelet-activated neutrophils (*via* PNAs) in islet-associated NETosis (in NOD mice) and *disease progression* to T1D-onset (in both NOD mice and humans) (15).

From a therapeutic perspective, T1D was prevented by treatment of adult NOD mice with a broad-acting inhibitor of PADs, which citrullinate proteins. Of these PADs, PAD4 citrullinates nuclear histones, a critical step in NETosis (57). This report clearly established the importance of citrullination in T1D autoimmunity, albeit without defining a role for NETs. In contrast, inhibitors that specifically target enzymatic products of neutrophils/NETosis (NE, MPO) or that neutralize NET/histone toxicity have had variable outcomes on T1D incidence (15, 44, 45), with most approaches failing to prevent T1D onset (15, 44). However, treatment of NOD mice from a very young age with NE inhibitors significantly reduced T1D incidence by dampening islet inflammation (45). Such interventions at the very onset of autoimmunity in humans are inevitably unrealistic. Therapies targeting NETs/neutrophil products in combination with short-term protocols targeting T cells (58, 59) may therefore provide more robust prevention of T1D in NOD mice and a basis for an improved clinical strategy for impeding T1D pathogenesis.

## T1D Is a Chronic Disease of Both the Endocrine and Exocrine Pancreas

Involvement of the exocrine pancreas in the pathogenesis of T1D, hypothesized long ago (60), is currently supported by documented reduction of pancreatic volume (61–63) and weight (64, 65), tissue damage and infiltration (16, 17, 66, 67) and subclinical impaired exocrine function (68–71). Interestingly, many of these exocrine abnormalities arise during pre-symptomatic T1D stages (17, 61, 64–67, 69–71), in parallel with the silent progression of endocrine dysfunction. These properties underpin the novel concept that T1D is a chronic disease of both the endocrine and exocrine pancreas and is thus an autoimmune or immune -mediated organ-specific disease, seemingly driven by a unique pathogenic process.

### Role of Neutrophils and NETosis in the Pathogenesis of T1D-Associated Pancreas Exocrine Disease

In parallel with the mild neutropenia and IFN-associated gene signature in neutrophils that accompanies T1D pathogenesis (16, 17), neutrophils infiltrate the exocrine tissue of the pancreas (16, 17, 67). This neutrophil influx appears very early, during the pre-symptomatic stages of T1D, at the time when islet autoantibodies are detectable, and persists afterwards (17). Notably, a fraction of pancreas-infiltrating neutrophils has been shown to extrude NETs (17), a known mechanism of tissue injury (72). Neutrophils may therefore act as innate immune effectors of exocrine and endocrine pancreas tissue injury in pre-T1D, with the formation of PNAs contributing to neutrophil activation, neutrophil recruitment to the pancreas and NETosis (15, 17).

### Innate and Adaptive Immunity in the Course of T1D Endocrine and Exocrine Pancreatic Disease

How does the accumulating evidence for neutrophils in the exocrine pancreas during T1D pathogenesis fit with the current knowledge of the natural history of the disease? The conventional paradigm for T1D identifies a predisposed genetic background, where some yet unidentified environmental factors induce an autoimmune response, marked by the appearance of islet-specific autoantibodies (single, then multiple), the progressive decline of beta cell mass due to autoreactive T cells (3, 73), diminishing beta cell function, finally ensuing in hyperglycemia and clinical T1D (74). Interestingly, in this model, the measurable immune markers, autoantibodies and autoreactive T cells, represent an expression of adaptive immunity towards endocrine pancreas. In contrast, there are no signs of an adaptive immune response against the exocrine pancreas.

**Figure 2:**
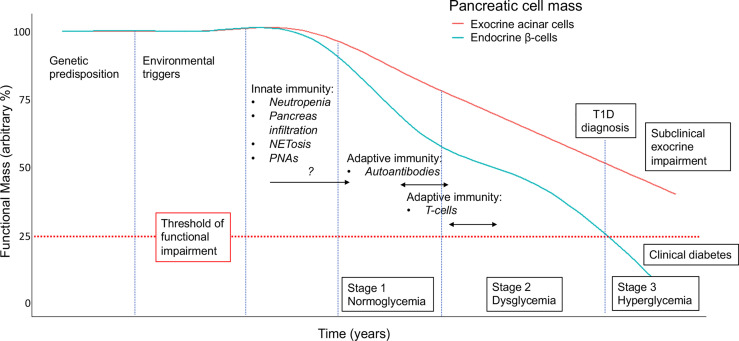
Proposed model for the contribution of neutrophils/NETs to the natural history of the decline in pancreatic endocrine (i.e., beta cell) and exocrine (acinar) function in T1D. Staging, as defined in (74), with modifications and incorporating assumptions based on available clinical and preclinical data. An arbitrary threshold is set at 25% to indicate functional pancreatic insufficiency. NETosis, neutrophils releasing extracellular traps; PNAs, platelet-neutrophil aggregates.

Conversely, neutrophil abnormalities are detectable very early in the course of T1D: neutropenia, pancreatic infiltration and elevated circulating PNAs are detectable in pre-symptomatic islet autoantibody-positive individuals (15, 17), while the IFN-gene oriented signature in peripheral neutrophils is found even prior to seroconversion (17). These observations are consistent with a precocious activation of innate immunity during T1D natural history, possibly before the activation of adaptive (auto)-immunity. In such a scenario, exocrine pancreas seems to be damaged by native immunity (i.e., neutrophils) rather than by adaptive immunity (e.g., possible bystander T cells (67)), while the endocrine pancreas is affected by both innate immunity (neutrophils) and adaptive immunity (autoreactive T cells). This may account for the different functional outcomes for the two pancreatic compartments in T1D. While endocrine dysfunction progresses to beta cell failure and clinical diabetes, the impairment of exocrine function remains subclinical, measurable in terms of reduced trypsinogen (71), fecal elastase, pancreatic amylase and lipase (68–70), but never reaching the degree of clinical insufficiency requiring pancreatic enzyme replacement therapy (summarized in **Figure 2**). How these two distinct processes influence each other is unknown, and remains a matter for future investigation of endotype interactions within T1D (9).

## Discussion

Evidence from T1D studies in humans and NOD mice suggests that neutrophils potentially have multiple roles in the pathogenesis of T1D: (i) *indirectly* aiding the initiation and expansion of autoreactive T cells and promoting islet inflammation by degranulation and/or NETosis, (ii) *directly* participating as effectors of islet/beta cell damage *via* NETs/NET proteins (extracellular histones) (15) and (iii) *directly* injuring pancreatic exocrine tissue. During T1D progression, neutrophils are rarely identified in islets/insulitis, possibly due to NETosis, and are more prevalent throughout the exocrine pancreas (15–17). NET proteins are more readily identified than NETs in both the endocrine and exocrine pancreas (15–17). These findings may in part be due to the rapid degradation of NET DNA by pancreatic DNAse (14) and/or to phagocytosis by macrophages (75), an activity which may be exacerbated by islet-associated inflammation (insulitis). Furthermore, NET proteins are more frequently detected in human exocrine pancreatic tissue than in islets during T1D progression (16, 17), consistent with the accompanying atrophy of exocrine tissue and function (18, 61, 63–71). Finally, heterogeneity in circulating neutrophil/PNA profiles accompanies both pre-symptomatic stages of T1D autoimmunity and T1D-onset in humans and NOD mice. Thus, the neutrophil signature in a subset of T1D and T1D-prone humans lends support to a neutrophil-associated T1D endotype.

## Author Contributions

CJS, AP, and EB wrote the manuscript; CRP provided intellectual input and edited the manuscript; SKP reviewed the manuscript; RF and SKP contributed the data in **Figure 1**. EB contributed **Figure 2**. All authors contributed to the article and approved the submitted version.

## Funding

This work was funded by an Innovation Research Grant to CJS from the Juvenile Diabetes Research Foundation (JDRF)Australian T1D Clinical Research Network (3-SRA-2018-602-M-B). AP was supported by a Juvenile Diabetes ResearchFoundation Advanced Postdoctoral Fellowship (3-APF-2019-744-AN). EB was supported in part by a Fondazione ItalianaDiabete (FID) research grant.

## Conflict of Interest

The authors declare that the research was conducted in the absence of any commercial or financial relationships that could be construed as a potential conflict of interest.

## Publisher’s Note

All claims expressed in this article are solely those of the authors and do not necessarily represent those of their affiliated organizations, or those of the publisher, the editors and the reviewers. Any product that may be evaluated in this article, or claim that may be made by its manufacturer, is not guaranteed or endorsed by the publisher.
